# Application of CuNPs and AMF alleviates arsenic stress by encompassing reduced arsenic uptake through metabolomics and ionomics alterations in *Elymus sibiricus*

**DOI:** 10.1186/s12870-024-05359-z

**Published:** 2024-07-13

**Authors:** Mansour K. Gatasheh, Anis Ali Shah, Muhammad Kaleem, Sheeraz Usman, Shifa Shaffique

**Affiliations:** 1 Department of Biochemistry, College of Science, King Saud University, P.O.Box 2455, Riyadh, 11451 Saudi Arabia; 2 Department of Botany, Division of Science and Technology, University of Education, Lahore, Pakistan; 3 College of Agriculture & Life Science, School of Applied Biosciences, Kyungpook National University, 80 Daehak-ro, Buk-Gu, Daegu, 41566 Korea

**Keywords:** Arsenic toxicity, Nanoparticles, Oxidative stress, Stress markers, Stress mitigation

## Abstract

Recent studies have exhibited a very promising role of copper nanoparticles (CuNPs) in mitigation of abiotic stresses in plants. Arbuscular mycorrhizae fungi (AMF) assisted plants to trigger their defense mechanism against abiotic stresses. Arsenic (As) is a non-essential and injurious heavy-metal contaminant. Current research work was designed to elucidate role of CuNPs (100, 200 and 300 mM) and a commercial inoculum of *Glomus* species (Clonex^®^ Root Maximizer) either alone or in combination (CuNPs + Clonex) on physiology, growth, and stress alleviation mechanisms of *E. sibiricus* growing in As spiked soils (0, 50, and 100 mg Kg^− 1^ soil). Arsenic induced oxidative stress, enhanced biosynthesis of hydrogen peroxide, lipid peroxidation and methylglyoxal (MG) in *E. sibiricus*. Moreover, As-phytotoxicity reduced photosynthetic activities and growth of plants. Results showed that individual and combined treatments, CuNPs (100 mM) as well as soil inoculation of AMF significantly enhanced root growth and shoot growth by declining As content in root tissues and shoot tissues in As polluted soils. *E. sibiricus* plants treated with CuNPs (100 mM) and/or AMF alleviated As induced phytotoxicity through upregulating the activity of antioxidative enzymes such as catalase (CAT) and superoxide dismutase (SOD) besides the biosynthesis of non-enzymatic antioxidants including phytochelatin (PC) and glutathione (GSH). In brief, supplementation of CuNPs (100 mM) alone or in combination with AMF reduced As uptake and alleviated the As-phytotoxicity in *E. sibiricus* by inducing stress tolerance mechanism resulting in the improvement of the plant growth parameters.

## Introduction

Arsenic (As) pollution has become an international agricultural, health and environmental issue because As is one of the most injurious heavy metals that may cause cancer and mutagenesis [1]. Natural geochemical progressions and numerous anthropogenic activities cause As pollution in an area [2]. Plants growing in As contaminated areas show poor physiochemical, growth and developmental attributes. Feeding on As contaminated foliage, flowers and fruits perhaps cause health hazards to humans, animals, and birds [3]. Factors affecting bioavailability and phytotoxicity of As include As species (As (V) and As (III)), concentration, plant species and mycorrhizal microbes in that area [4]. The inorganic forms of As (As (V)), mostly existing as free anions, are more injurious as compared to organic (As (III)) ones which may develop complex with proteins and phytochelatins [5]. The exact mode of action for As uptake, translocation and bioaccumulation in plants is still unclear. Additionally, physiochemical, and omics mechanisms to detoxify and mitigate As phytotoxicity may vary in different plant species [6]. In general, the cytotoxic methylglyoxal (MG) synthesized in metal stressed plants hinders seed germination, photosynthetic activity, DNA activation, and biomass production. Alternatively, the glyoxalase (Gly) defense system of plants comprising glyoxalase I and glyoxalase II detoxifies MG into D-lactate to tolerate abiotic stress [7]. Plants are capable of alleviating heavy metal stress through activating their defense system. Plants synthesize a range of biomolecules to mitigate metal induced phytotoxicity. Additionally, plants engage antioxidant enzymes such as ascorbate peroxidase, catalase, superoxide dismutase and glutathione reductase and non-enzymatic antioxidants including phytohormones, nitric oxide, proline, nutrients, phytochelatins, glutathione to alleviate abiotic stresses [8, 9].

Nano-agriculture comprises the use of nanoparticles (NPs) based fertilizers for crop production and stress alleviation. The higher efficiency of nano fertilizers is attributable to higher density in the reactive zones of nanoparticles [10]. Copper (Cu) is an essential micronutrient which plays a pivotal role in several physiochemical activities of plants for example plant mitochondrial respiration, redox regulation, stress tolerance, photosynthetic activity, protein synthesis and tissues formation [11, 12]. Presently, the application of copper nanoparticles (CuNPs), having higher conductivity as compared to other metallic nanoparticles, has tremendously increased in crop production [13, 14]. Farmers are applying CuNPs based growth regulators, insecticides, weedicides, fungicides, fertilizers, additives for soil remediation [15, 16]. According to [17], the use of CuO-NPs may reach about 1600 tons during 2025. Researchers have observed beneficial and harmful effects of CuNPs in various plant species. [18] have revealed the growth promoting effects of CuNPs when applied to lettuce plants. Controversially, [19] revealed that CuNPs induced toxicity decreased chlorophyll content, photosynthesis, stomatal conductivity. So, it becomes mandatory to evaluate the beneficial and harmful influences of CuNPs on growth and development of different plant species.

Arbuscular mycorrhizal fungi (AMF) belonging to the phylum Glomeromycota, commonly exist in the rhizospheric area of metal spiked soils and assist in bioremediation [20]. *Glomus* species which belong to the family *Glomeraceae* are the most important AMF [21]. The quickly germinating spores of AMF members of *Glomeraceae*, commonly existing in rhizospheric areas of poor-quality soils, may easily develop symbiotic relationships with neighboring plant roots [22]. Consequently, application of AMF is an economical and ecofriendly approach for bioremediation of metal contaminated soils [23]. Plant associated AMF restrain the mobility of phytotoxic metal ions towards plant roots. AMF may adjust the metal concentration in plants because mycelia, spores and vesicles of AMF perform as an operative sink or chelators of heavy metals [24]. The symbiotic association of plants with AMF enhance their stress tolerance system to grow in metal spiked soils. In return, the hyphae of AMF provide mineral nutrients to assisted plants in exchange for photosynthates. Phytohormones and metabolites synthesized by AMF improve plant growth [25, 26]. Hence, AMF directly mitigates plant stress or enhances phosphorus (P) uptake which indirectly detoxifies metals and enhances plant stress tolerance [27, 28]. In some cases, AMF reduce the uptake of heavy metals including Cu, Mn, Cd and Zn. While in other associations, AMF augments uptake of metal ions in plants [29, 30]. Additionally, AMF are capable of regulating the expression level of genes related to As and P transportation in plants [31].

Application of CuNPs may induce metal toxicity in some plants. However, AMF inoculation alleviates metal stress through improving the antioxidative system leading to reduced biosynthesis of ROS besides augmenting the expression of antioxidant responsive genes [32, 33]. The elevated level of metallothionein genes and Cu-transport genes in AMF inoculated plants decreased Cu uptake and translocation [34, 35]. Besides, higher expression level of organic acid metabolism-associated genes in AMF assisted plants enhanced the biosynthesis glomalin like organic acids which reduce bioaccumulation of Cu [36].

*Elymus sibiricus* (Siberian wildrye) which belongs to the family *Poaceae* is a self-pollinating perennial grass. It is a stress tolerant grass and thrives best in sandy coastlines, wet pastures, open jungles, mountainous slopes and basins from 33,00 to 13,200 ft. This grass improves the ecosystem of the natural grasslands and is extensively cultured as hay and pasture owing to its higher produce, robust tillering dimensions, better nutrition, easy cultivation and outstanding adaptability to temperate and tropical climatic zones [37, 38]. Despite numerous studies on the metal stress alleviation potential of CuNPs and AMF when applied separately in various crop plants [39]. However, according to our knowledge, there is no report about the stress ameliorative role of copper nanoparticles and AMF combinations on *E. sibiricus* subjected to As stress. It is assumed that the combined application of CuNPs and AMF for As stress mitigation will be an effective, inexpensive, and enduring stratagem. Hence, during this study, Clonex^®^ was used due to its capability to increase P uptake and plant growth in plants in addition to its ability to enhance metal stress tolerance in plants. Consequently, it was hypothesized that CuNPs and AMF would improve physiochemical attributes, nutrition, growth, and stress tolerance of As stressed *E. sibiricus* plants. Therefore, the aim of this research work was to observe the advantageous effects of CuNPs and AMF on physiochemical activities, photosynthesis, antioxidative system, and growth of *E. sibiricus* plant under As stress. Results of the current research will unveil the mechanisms involved in reduction of As uptake and phytotoxicity besides advancement of the approaches to continue sustainable crop production in As polluted soils.

## Materials and methods

### Synthesis of copper nanoparticles

The chemicals used in the present study are copper sulphate (CuSO_4_.5H_2_O) and sodium hydroxide (NaOH), see Table 1. Double distilled de-ionized water was used for making the solutions. Chemicals in-use (see Table 1) were of high purity and of analytical grade so there was no need for further purification. They were used in the synthesis of this work as they were received.

**Fig. 1:**
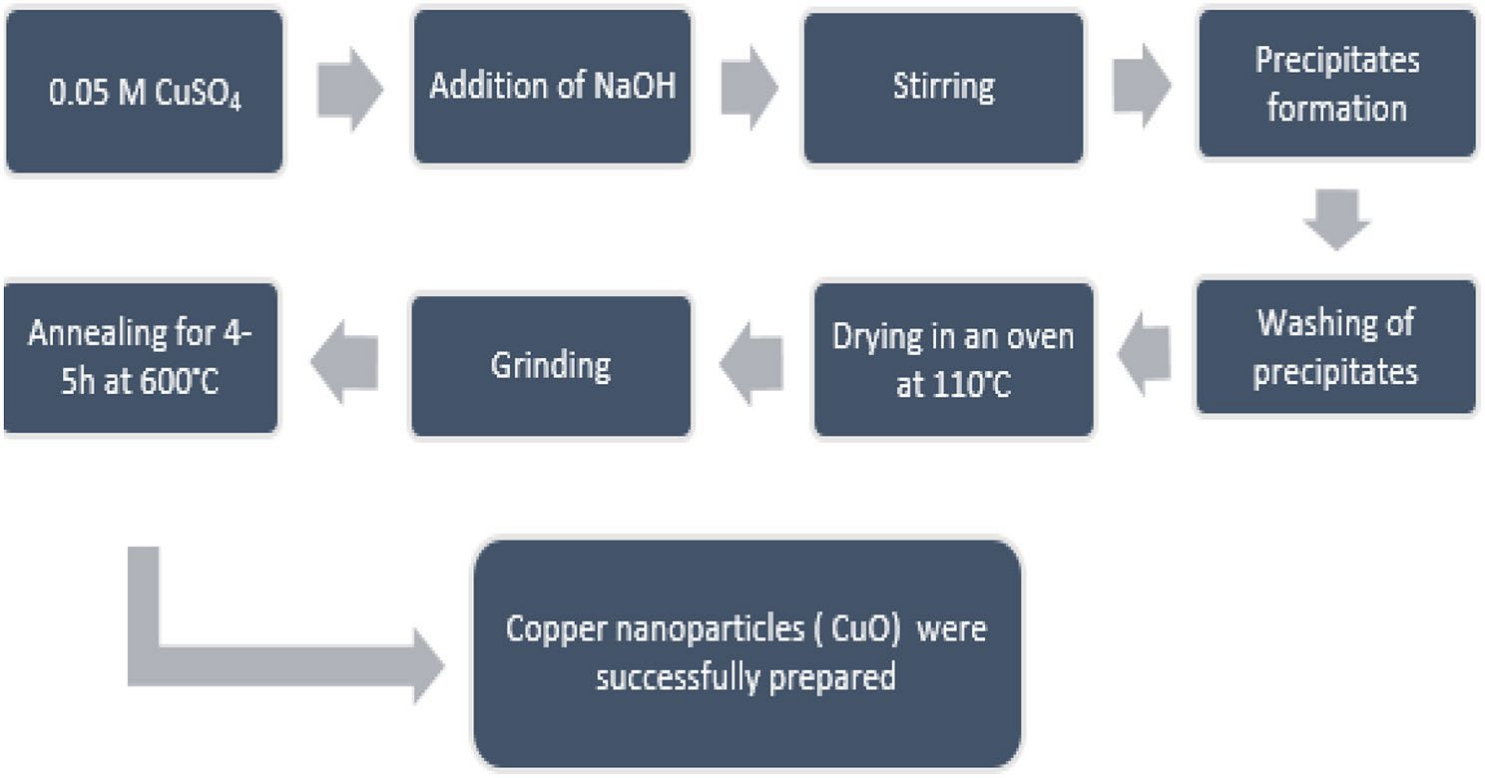
Flow sheet diagram of synthetic route for preparation of copper nanoparticles by co-precipitation method

**Fig. 2:**
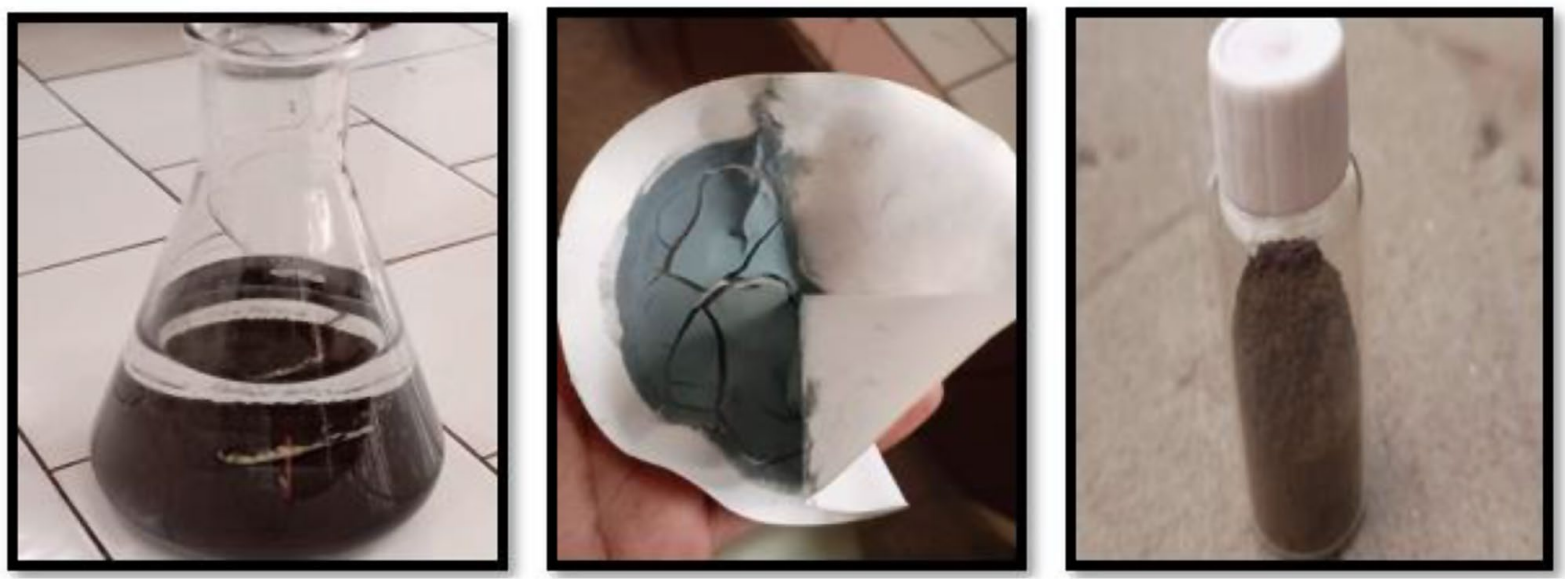
Synthesis of CuO-NPs

The co-precipitation process was used for producing copper nanoparticles. This process involves dissolving precursor solutions with the necessary molarity in distilled water, such as CuSO_4_.5H_2_O and NaOH. CuSO_4_.5H_2_O was dissolved in a conical flask to a concentration of 0.05 molar. The equations described above are used to create a 0.1 molar NaOH solution in a different conical flask. Then, 0.1 molar NaOH solution was dropped one at a time into the conical flask containing the CuSO_4_.5H_2_O solution. Slow addition and constant stirring were used throughout the procedure. The solution turns from blue to black with addition of 0.1 M NaOH solution. Metal hydroxides precipitate after being added to the base drop by drop. These precipitates are then rinsed via distilled water after being filtered using filter paper. Washing the precipitates was done to get rid of any sulphate that was left behind. After drying, the crystals were ground into a fine powder. The weight of the sample was used to compute the actual yield product. Muffle furnace annealing was done for 4–5 h at 600 °C. CuO nanoparticles are created when the Cu(OH)_2_ was annealed. The weight of CuO-NPs was once again measured after annealing (Figs. 1 and 2). Equation shows that the final result, copper nanoparticles, was successfully created after the whole procedure [40].

1 $$\text{C}\text{u}\text{S}{\text{O}}_{4}0.5{\text{H}}_{2 }{\text{O}}_{\left(\text{a}\text{q}\right)}+\text{N}\text{a}\text{O}\text{H} \to 2 {\text{N}\text{a}}_{2}\text{S}{\text{O}}_{4 \left(\text{a}\text{q}\right)}+\text{C}\text{u}{\left(\text{O}\text{H}\right)}_{2 \left(\text{s}\right)}+5 {\text{H}}_{2}{\text{O}}$$

### Experimental design and layout

A completely randomized design experiment with three replicates was performed at Botanical Garden, University of Education, Lahore, Pakistan. In whole the experiment consisted of 72 pots containing 8 Kg of soil. Seeds were obtained from Punjab Seeds Cop. Lahore. 10–12 healthy seeds were sown in each pot, thinning was done on second week of germination and only 4–5 plants were maintained in every pot. Arsenic contamination was given at the rate of 50 and 100 mg Kg^− 1^. CuNPs (Control, 100, 200 and 300 mM) were applied by foliar spray while mycorrhizal were also tested through soil amendments (either included or excluded). One plant from each pot was uprooted to mount growth parameters (root and shoot length), on fourth week of treatments.

### Chlorophyll contents

0.5 g fresh leaf sample was homogenized in 80% acetone solution. After filtration the extract was mounted in UV-VIS spectroscope at different wavelengths 645 and 663 nm to read OD [41]. Total chlorophylls were calculated by sum of chl *a* and chl *b.*

### Chlorophyll a fluorescence and PI_ABS_

Chlorophyll a fluorescence was estimated in field conditions using automated OS30P^+^ ADC Bioscientific fluorometer. Fully developed young leaves were dark adapted for 20 min using clips. Following that dark-adapted leaves were given saturation pulse at 3500 µmol and modulation light intensity set to 40%. Various biophysical parameters were estimated such as F_v_/F_m_ and PI_ABS_. The performance index was expressed on absorbance basis [42].

### Assessment of electrolyte leakage (EL)

Estimation of inorganic ions leakage from foliage was analyzed according to [43]. For this purpose, 10 fresh leaf segments were submerged in 20 mL of boiling deionized water present in a test tube. The initial electron conductivity (EC1) was evaluated. Afterwards, a tube containing solution was placed over a water bath at 50ºC for 0.5 h to estimate EC2. Then, solutions containing tubes were placed over a water bath at 100ºC for 10 min to estimate EC3. The value of EL from leaf sections was computed according to the following formula:

$$EL \left(\%\right) =\left[\right(EC2-EC1)/(EC3\left)\right] \times 100$$

### Assessment of oxidative stress markers

#### Superoxide anion

The level of superoxide radical (O_2_^•−^) was estimated by homogenizing a 4 g foliage sample in 6 mL of 3% trichloroacetic acid. The resulting solution was subjected to centrifugation at 10,000×*g* for 22 min. The reaction solution was prepared by adding 1 mM hydroxylamine hydrochloride in 2 mL supernatant and 2 mL of 50 mM potassium phosphate buffer at pH 7.1. The optical density of the reaction solution was observed at 530 nm to estimate O_2_^•−^ level by comparing with the standard curve [44].

#### Lipid peroxidation

Fresh leaves from each replicate were collected, weighed (0.5 g), and thoroughly mixed with 0.1% trichloroacetic acid (5 mL) in a pre-chilled pestle and mortar. The extracts were put into conical tubes. The extracts were centrifuged at 12,000 g for 15 min at 4 °C. Potassium phosphate buffer (0.5 mL, pH7), potassium iodide (1 mL), and supernatant (0.5 mL) from centrifugation were placed in test tubes. The mixture was vortexed and the absorbance at 390 nm was measured with a UV/VIS spectrophotometer [45].

#### Hydrogen peroxide (H_2_O_2_)

Biosynthesis of H_2_O_2_ was analyzed with the help of a spectrophotometer following reaction with potassium iodide (KI). The reaction solution was prepared by adding 1 mL of 100 mM K-phosphate buffer, 4 mL reagent (1 mM KI w/v in fresh double-distilled water H_2_O) and 1 mL of 0.1% trichloroacetic acid (TCA) leaf extract supernatant placed in darkness for 60 min. Afterwards, absorbance of the blank containing 0.1% TCA and reaction mixture was observed at 390 nm. Quantity of H_2_O_2_ was analyzed by comparing it with a standard curve of H_2_O_2_ [46].

### Estimation of the activity of antioxidative enzymes

For extraction of enzymes, 2 g fresh plant sample was homogenized in an ice chilled mortar in presence of 100 mg polyvinylpolypyrrolidone (PVPP) and 40 mL of the mixture containing 0.1 mM EDTA and 50 mM of K-phosphate buffer at pH 7.6. For glutathione reductase (GR) estimation, 10 Mm of β-mercaptoethanol was added in the solution.

### Superoxide dismutase (SOD) activity

Superoxide dismutase can impede the reduction of nitro-blue tetrazole (NBT) through photochemically created superoxide radicals. Activity of this enzyme was analyzed according to [47] by assessing the SOD quantity required to hinder the half reduction rate of NBT at room temperature.

### Catalase (CAT) activity

For evaluation of CAT activity, 100 µL of the extract was mixed with 50 mM potassium phosphate buffer (pH 7.0) and 10 mM of H_2_O_2_ at room temperature. Enzymatic activity was assessed against blank (lacking enzyme extract) at 1 min intermission for 3 min through observing the reduction in H_2_O_2_ consumption at 240 nm [48].

### Ascorbate peroxidase (APX) activity

The reaction was analyzed in a 1 mL solution containing 2.5 mM H_2_O_2_, 1 M sodium ascorbate and 80 nM of potassium phosphate buffer. Estimation for oxidation ratio of ascorbate, H_2_O_2_ was supplemented to initiate the reaction and the reduction of absorbances was evaluated for 1 min at 290 nm [49].

### Glutathione reductase (GR) activity

For estimation of GR activity absorbance of reaction mixture (3 mM, 1 mM of GSSG, 50 mM of buffer Tris–MgCl_2_, 0.3 mM nicotinamide adenine dinucleotide phosphate and 25 µL of enzyme) was perceived at 340 nm at room temperature [50]. Enzymatic activity was assessed with the primary rate of the reaction and the molar extinction coefficient of NADPH.

### Glutathione peroxidase (GPX)

Activity of GPX was examined by employing a microplate reader and observing spectrophotometric absorbance [51]. The 500 µL reaction mixture used for this purpose included 20 µg of extracted proteins, 1 mM NADPH, 1 µ of glutathione reductase, 1 mM EDTA, 2 mM glutathione, 2 mM t-butyl hydroperoxide and 100 mM sodium phosphate buffer at pH 7.0. The rate of NADPH oxidation was analyzed at 340 nm over a time period of 15 min.

### Glutathione–S-transferase (GST)

The reaction mixture used to evaluate the GST activity included 1 mM 2,4 dinitrochlorobenzene, 20 mM glutathione, 10 µL plant extract and 0.1 M phosphate buffer at pH 7.5. The spectrophotometric value was observed at 340 nm via employing an extinction coefficient of 9.6 mM^–1^ cm^–1^ [52].

### Assessment of non-enzymatic antioxidant

#### Ascorbic acid (AsA)

The AsA present in a 50 mg frozen foliage sample was extracted with the help of 6% trichloroacetic acid. This extract (8 mL) was homogenized with 4 mL dinitrophenylhydrazine (2%) and 2 drops of 10% thiourea solution (in 70% ethanol) and boiled for 15 min in bain-marie. After cooling, 10 mL sulfuric acid (80% v/v) was mixed in it at 0 °C to observe a spectrophotometric value at 530 nm according to [53]. The obtained values were compared with a standard curve of known ascorbic acid solutions.

#### Glutathione (GSH)

For quantification of glutathione in reduced form, 2 g foliage sample was vortexed with 20 mL metaphosphoric acid (5%) at freezing temperature. This mixture was centrifuged at 15,000*g* for 0.5 h at 4 °C and supernatant was observed at 412 nm and compared with the standard curve of glutathione [54]. Following the elimination of GSH through 2-vinylpyridine derivative, the quantity of glutathione disulfide (GSSG) was estimated. Amount of GSH was computed through deduction of glutathione disulfide (GSSH) quantity from total glutathione.

### Estimation of as uptake in root and shoot of *E. sibiricus*

The sample digestion was performed by taking 0.1 g root and shoot samples in test tubes, added in it 10 mL of HNO_3_ and left over for night. Following that 8 mL of HClO_4_ was added to the mixture. This mixture was transfer to digestion flask and heated on hot plate until fumes formation stopped. A colorless solution formed at the end confirmed completion of digestion. Cool the solution and add 20 mL of distilled water. After filtering, the solution was analyzed for arsenic heavy metal using an atomic absorption spectrophotometer [55].

### Statistical analysis

DSAASTAT ver. 1.514 was used for ANOVA test. Tukey’s HSD mean compare test was performed at *p* < 0.05. R software was used for Pearson’s correlation and principle component analysis test. MS excel was used to make graphs.

## Results

### Characterization techniques

Then the dried powder was collected and characterized by Fourier-transform infrared spectroscopy (FTIR), scanning electron microscopy (SEM-EDS). The morphology and chemical composition of the copper nanoparticles were studied by scanning electron microscope (SEM, TESCAN, Model: VEGA3 LMU). The functional groups present could be analyzed by FT-IR while presence of impurities could be determined by EDX.

#### Scanning electron microscopy (SEM)

Scanning electron microscopy (SEM) provides morphological analysis with direct visualization, The SEM image was used to assess the true size of nanoparticles. The methods based on electron microscopy have several benefits in terms of morphological and sizing analyses, but they give little insight into the size distribution and genuine population average. Before being coated with a conductive metal, such as gold, using a sputter coater, a nanoparticle solution should be first turned into a dry powder and deposited on a sample holder for SEM analysis. A focussed fine electron beam was then utilized to scan the material. The secondary electrons released from the sample surface are used to determine the surface properties of the sample. The polymer may be harmed by the electron beam, and the nanoparticles must be able to tolerate vacuum. The findings from dynamic light scattering are similar to the mean size found by SEM. Furthermore, these methods require a lot of time and money, and they typically need for additional data on the distribution of sizes. Most nanoparticles are approximately 50 nm in size, which was consistent with results from particle size analysers. The SEM graph also demonstrates the sheet- or rod-like form of the copper oxide nanoparticles.

**Fig. 3:**
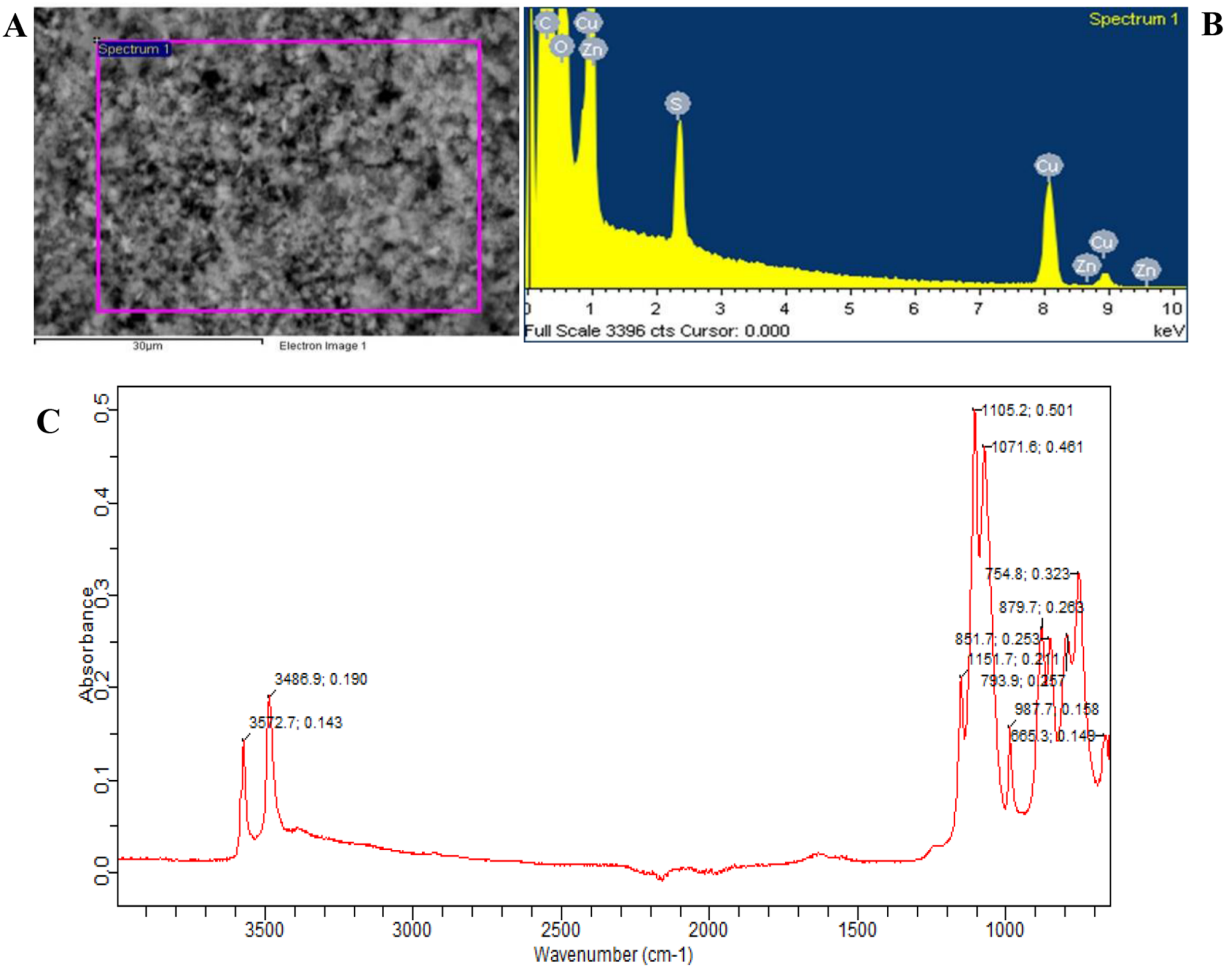
Characterization of copper oxide nanoparticles (CuNPs). (**A**) Scanning electron micrograph (SEM) for CuNPs, (**B**) Energy dispersive X-Ray (EDX) graph for CuONPs, (**C**) Fourier transform infrared (FTIR) spectra for CuNPs

Figure 3A depicts the copper oxide nanoparticles as seen via a scanning electron microscope (SEM) at a reduced magnification. Although the particles are slightly clumped together, most of them are in the nanometre range. Weak physical forces are what keeps the particles together. This indicates that copper oxide created using copper sulphate as the starting material could generate particles with size in the nanometre range, and that the particle separation was excellent and that the preparation procedure was extremely effective since particles were formed here with size in the nano range. As a result, the SEM picture in Fig. 3A that depicts a uniform distribution of spherical CuO nanoparticles was used to disclose the surface morphology of the manufactured CuO nanoparticles.

#### Energy dispersive X-ray (EDX)

Using Energy Dispersive X-Ray (EDX) spectroscopy, the elemental makeup of the produced nanoparticles was measured. The EDS spectra of the nanoparticles created using sodium hydroxide as a reducing agent was shown in Fig. 3B. At 600 °C, CuO-NPs were annealed. CuO-NPs were used in the EDX study at a 10 keV energy level. The results and EDX analysis of copper oxide nanoparticles show that the nano powders are almost stoichiometric. This spectrum provided proof that Cu, O, S, Zn, and C exist. The peak at 0.2 keV was associated with the binding energy of carbon (CKa), whereas the peaks at 0.5, 0.8, and 0.9 keV are associated with Oka, CuKa, and ZnKa, respectively. In addition, a peak for carbon (CKa) at 3.7 keV has also been observed. With the exception of a slightly elevated signal for carbon, the measured EDX spectra of Cu nanoparticles was identical to that previously reported by Kooti and Matouri. The samples’ carbon and oxygen peaks confirmed the existence of stabilizers made of carbon. According to the weight% of various components, oxygen was 34.81% and copper was 53.12%. Sulphur, carbon, and zinc are the additional components; their existence was represented by 6.31, 2.21 and 3.55%, respectively. The proportion of each element found in the sample was shown in Table 2. The contamination from the carbon tape used in the study may be blamed for the presence of elements like C, S, and Zn, while the contamination from Zn foil was to blame for the presence of Zn. The oxidation of copper powder was to blame for the oxygen seen in the spectra.

#### Fourier transform infrared (FTIR) spectroscopy

To determine the various functional groups contained in the synthesized samples, FT-IR spectroscopy was used. It truly demonstrates that the functional groups for which engineered material was produced are present. These findings also provide a useful way to compare our obtained data to earlier referenced standard data in the literature.

Numerous functional groups may bond to copper (II) oxide. N-H has two peaks in the aforementioned finding, both at 3572.7 cm^− 1^. A C-O group may be found at wave number 1071.60 cm^− 1^. These findings are essentially consistent with data from the literature. Peaks from 754.8 to 851.7 cm^− 1^, meanwhile, are a result of Cu-O stretching modes. At these locations, there was a significant absorption band associated with the vibrations of the Cu-O functional group. This verified the existence of CuO particles at the nanoscale. The majority of the absorption peaks, which may be attributed to copper atoms and O-H bending vibrations, are found in the 987.7 to 1151.7 cm^− 1^ range. Therefore, according to the FTIR results, the annealing process in this approach results in the synthesis of copper oxide compound together with the existence of Cu-O bonds that correspond to Cu-O stretching modes (Fig. 3C).

The work showed a promising and broadly applicable technique for chemically synthesizing copper oxide nanoparticles. Due to its straightforward procedure and inexpensive cost, this synthesis route was especially well suited for the large-scale production of CuO nanoparticles. Different methods, including scanning electron microscopy (SEM), energy dispersive x-ray (EDX) spectroscopy, and Fourier transform infrared (FT-IR) spectroscopy, were used to describe the thusly produced crystals. The production of scattered copper nanoparticle agglomerates was seen in the SEM pictures. The existence of elemental copper, some oxygen, and other contaminants are confirmed by the EDX analysis of copper nanoparticles. The generated nanoparticles’ primary functional group peaks may be seen using FT-IR. It was likely that the improvement of CuO nanocrystal synthesis processes and greater understanding of their characteristics will result in a significant breakthrough in their applications.

### Effect of CuNPs and mycorrhiza association on growth of *E. sibiricus* under arsenic stress

Growth parameters (root and shoot length) of *E. sibiricus* were hampered significantly when grown in 50 mg Kg^− 1^ (5.46 cm, 16.06 cm) and 100 mg Kg^− 1^ (4.87 cm, 14.34 cm) As contaminated soil, as equated with control plants (7.8 cm, 22.95 cm). Conversely, CuNPs foliar spray, at varying concentrations (100, 200 and 300 mM), alone and in combination with mycorrhizal soil amendments effectively improved growth of *E. sibiricus* plants in stress as well as non-stress conditions, as shown in Tables 3 and 4.

### Effect of CuNPs and mycorrhiza association on photosynthesis related attributes of *E. sibiricus* under arsenic stress

#### Chlorophyll contents

**Fig. 4:**
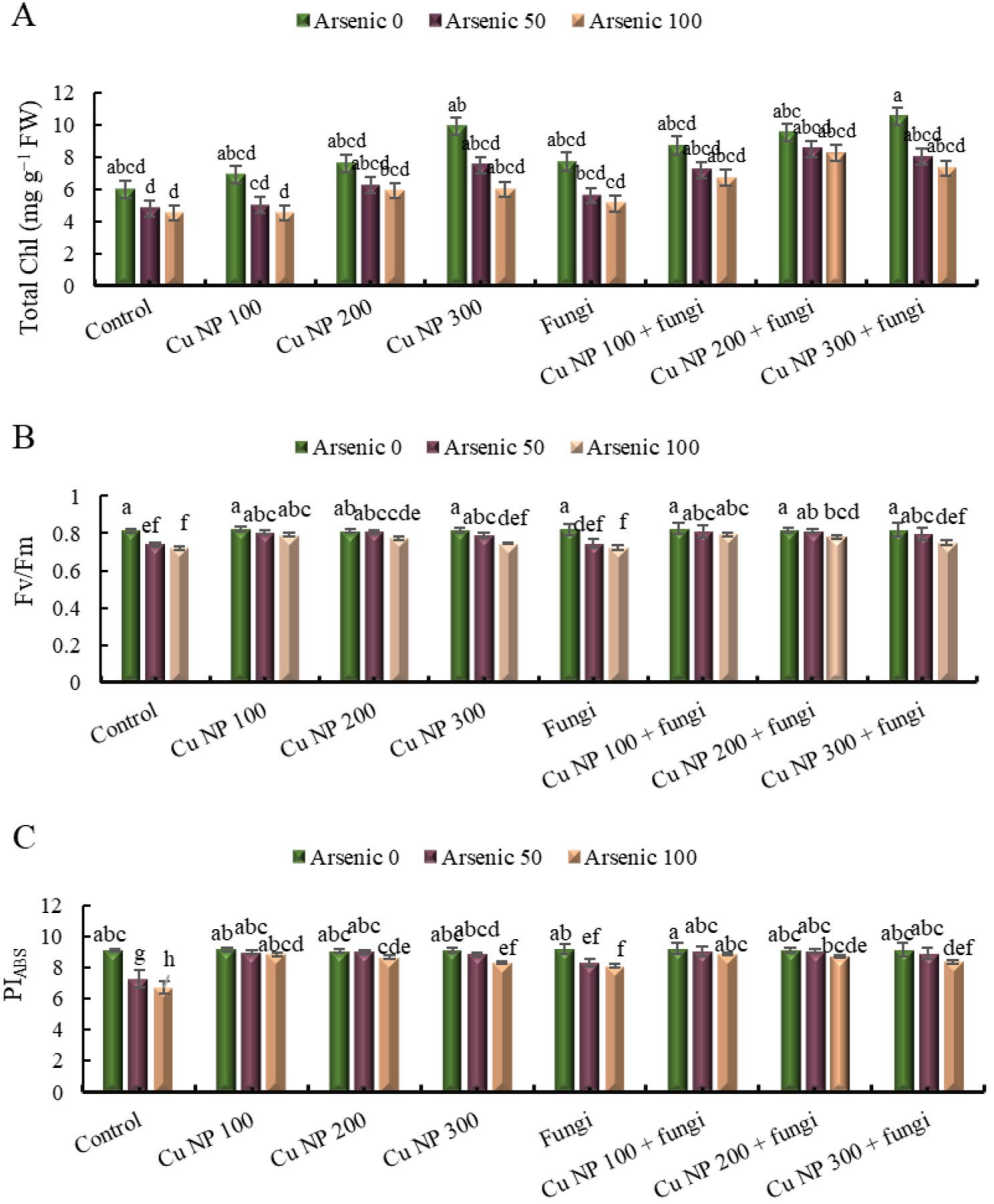
Effect of CuNPs and mycorrhiza association on (**A**) total chlorophyll, (**B**) quantum efficiency of PSII (Φ PSII) and (**C**) performance index of chlorophylls in *Elymus sibiricus* under arsenic stress. Graph bars represents mean value of three replicates. Error bars shows standard error and different letters above bars obtained after Tukey’s HSD test, represents mean values are significantly different at *p* < 0.05

The data provided outlines the effects of different treatments on chlorophyll levels in a certain experimental setup. The control group, which experienced no stress, maintained a chlorophyll level of 0%. However, when subjected to stress in the form of 50 and 100 mg Kg^− 1^ doses, the chlorophyll levels dropped by 30% and 38% respectively. Introducing copper nanoparticles at various concentrations showed mixed results: at 100 mM concentration, it led to a 23% increase in chlorophyll levels in the control group but resulted in decreases of -25% and − 38% when combined with arsenic doses of 50 and 100 mg Kg^− 1^ respectively. Similarly, at 200 mM concentration, it boosted chlorophyll levels by 40% in the control group, with smaller positive effects seen when combined with arsenic. The highest concentration tested, 300 mM, led to a substantial 98% increase in chlorophyll levels in the control group, with varying effects when combined with arsenic. Additionally, the introduction of arbuscular mycorrhizal fungi had notable impacts: in the control group, it increased chlorophyll levels by 43%, but showed reductions of -10% and − 23% when combined with arsenic doses of 50 and 100 mg Kg^− 1^ respectively. However, when combined with copper nanoparticles, particularly at higher concentrations, the arbuscular mycorrhizal fungi demonstrated synergistic effects, leading to significant increases in chlorophyll levels compared to either treatment alone (Fig. 4A).

### Quantum efficiency of PSII (Φ PSII) and performance index (PI)

As-stress dropped down Φ PSII significantly. Φ PSII reduced by 9 and 11% at 50 and 100 mg Kg^− 1^ arsenic contamination, respectively. The foliar spray of CuNPs and mycorrhizal amendments in soils revive the Φ PSII in As-stress plants as compared to stress only plants. Maximum Φ PSII was observed in control plants with 100 mM CuNPs foliar spray and mycorrhizal soil amendments. Similar findings were also observed for performance index of chlorophyll *a*, where drop down in PI by 50 mg Kg^− 1^ (20%) and 100 mg Kg^− 1^ (26%) arsenic soil contamination was very significant. In contrast to As-stress, foliar application of CuNPs and mycorrhizal soil amendments resulted in improved PI in control as well as stressed plants. Maximum PI was observed in plants treated with 100 mM CuNPs foliar spray along with mycorrhizal soil amendments. The Fig. 4B and C depicted that CuNPs application in combination with mycorrhizal soil amendments was more effective at low level (100 mM) as compared to high level (200 mM), in alleviation of As-stress.

### Effect of CuNPs and mycorrhiza association on stress markers of *E. sibiricus* under arsenic stress

#### Malondialdehyde (MDA)

**Fig. 5:**
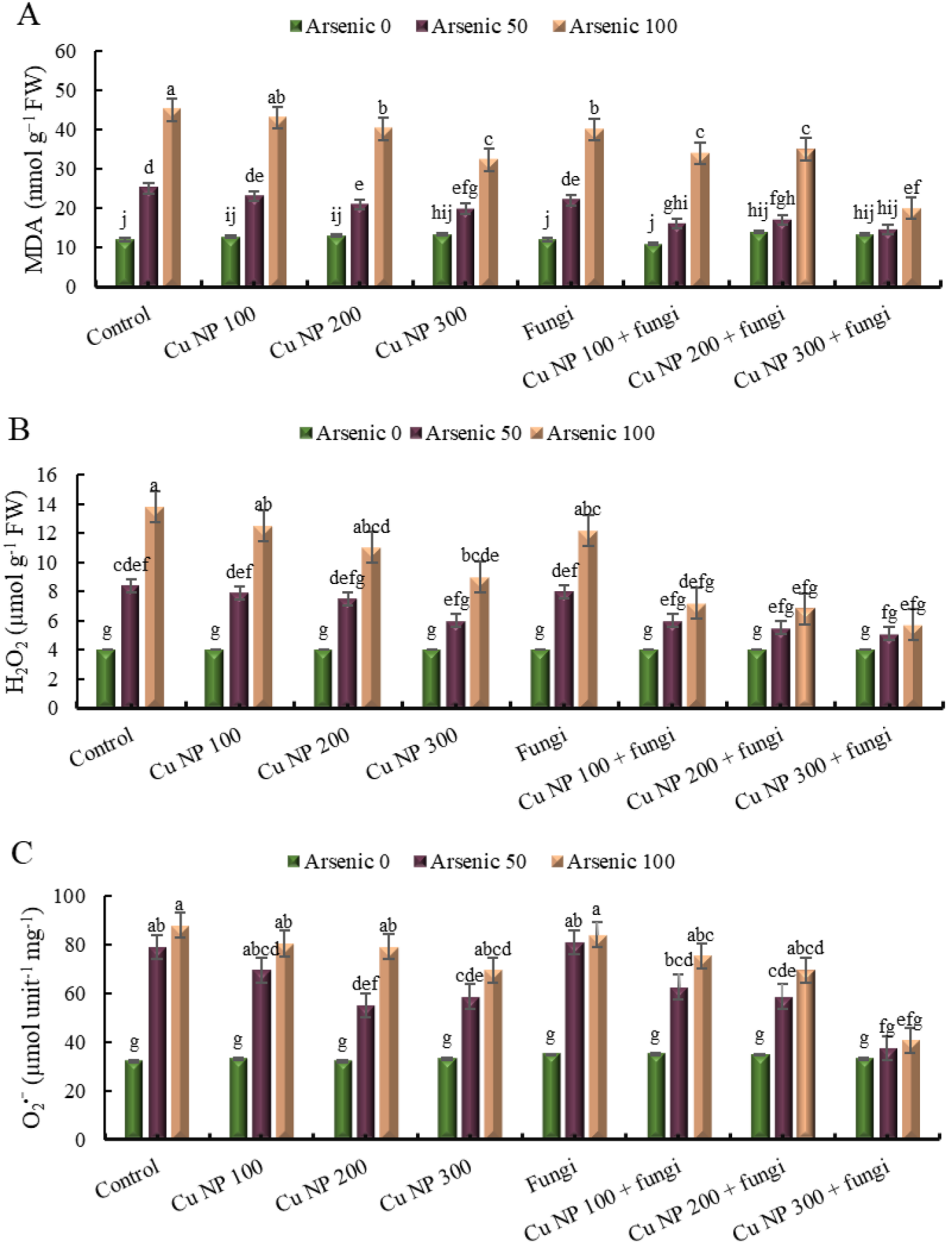
Effect of CuNPs and mycorrhiza association on (**A**) malondialdehyde, (**B**) hydrogen peroxide and (**C**) superoxide anions in *Elymus sibiricus* under arsenic stress. Graph bars represents mean value of three replicates. Error bars shows standard error and different letters above bars obtained after Tukey’s HSD test, represents mean values are significantly different at *p* < 0.05

The data provided outlines the effects of various treatments on a group labeled MDA. The control group, which experiences no stress, was set at 0%. When subjected to stress with 50 mg Kg^− 1^ of a certain substance, the response increases to 108%. Doubling the stress to 100 mg Kg^− 1^ results in a significant jump to 275%. Introducing copper nanoparticles at 100 mM concentration in the control group leads to a response of 7%. However, when arsenic was added at 50 mg Kg^− 1^ alongside these nanoparticles, the response escalates to 92%, and at 100 mg Kg^− 1^ of arsenic, it further increases to 258%. Similarly, with copper nanoparticles at 200 mM concentration, the control response was 8%, but with added arsenic at 50 mg Kg^− 1^, it rises to 74%, and at 100 mg Kg^− 1^, it jumps to 235%. The trend continues with copper nanoparticles at 300 mM concentration, where the control group shows a 12% response, but with added arsenic at 50 mg Kg^− 1^, it increases to 65%, and at 100 mg Kg^− 1^ of arsenic, it reaches 169%. Introducing arbuscular mycorrhizal fungi in the control group yields no response, but with added arsenic at 50 mg Kg^− 1^, the response was 83%, and at 100 mg Kg^− 1^ of arsenic, it rises to 233%. Interestingly, the addition of copper nanoparticles at 100 mM concentration alongside the fungi results in a negative response (-8%). However, when arsenic was added at 50 mg Kg^− 1^, the response becomes positive at 33%, and at 100 mg Kg^− 1^ of arsenic, it significantly increases to 183%. Similar patterns are observed with copper nanoparticles at 200 mM and 300 mM concentrations. These results suggest complex interactions between stressors and treatments, with some combinations exacerbating responses and others mitigating them (Fig. 5A).

#### Hydrogen peroxide (H_2_O_2_)

The control without stress shows a baseline of 0%, while the control with 50 mg Kg^− 1^ exhibits a 110% increase, and the control with 100 mg Kg^− 1^ shows a 245% increase. When copper nanoparticles at 100 mM concentration are introduced without arsenic, there was no change (0%), but when arsenic was added at 50 and 100 mg Kg^− 1^, there are increases of 98% and 213%, respectively. Similarly, copper nanoparticles at 200 mM and 300 mM concentrations show no change (0%) when introduced alone but result in increases when combined with arsenic. Arbuscular mycorrhizal fungi alone show no change (0%) in the control, but with arsenic at 50 and 100 mg Kg^− 1^, there are increases of 100% and 205%, respectively. When combined with copper nanoparticles at different concentrations, the effects vary, with some combinations showing decreases compared to arsenic alone. Overall, the data illustrate the complex interactions between different treatments and their effects on the group H_2_O_2_ under varied conditions (Fig. 5B).

#### Super oxide anions

The data represents the levels of O_2_^•−^ under various conditions. In the control group without stress, the level was recorded at 0%. However, when exposed to stress with 50 mg Kg^− 1^ of a certain substance, the level rose significantly to 148%. Further increase to 100 mg Kg^− 1^ resulted in a higher level of 175%. Introducing copper nanoparticles at 100 mM concentration in the control group notably decreased the level to 3%. When combined with arsenic at 50 mg Kg^− 1^, the level increased to 118%, and at 100 mg Kg^− 1^, it rose further to 152%. Similar patterns were observed with copper nanoparticles at 200 mM and 300 mM concentrations. Additionally, the introduction of arbuscular mycorrhizal fungi showed varying effects: in the control group, it resulted in a level of 9%, while with arsenic at 50 and 100 mg Kg^− 1^, the levels increased to 154% and 163% respectively. Combining arbuscular mycorrhizal fungi with different concentrations of copper nanoparticles exhibited diverse impacts on the levels of Super Oxide anions, showing both increases and decreases depending on the concentration of both substances (Fig. 5C).

#### Electrolyte leakage (EL)

**Fig. 6:**
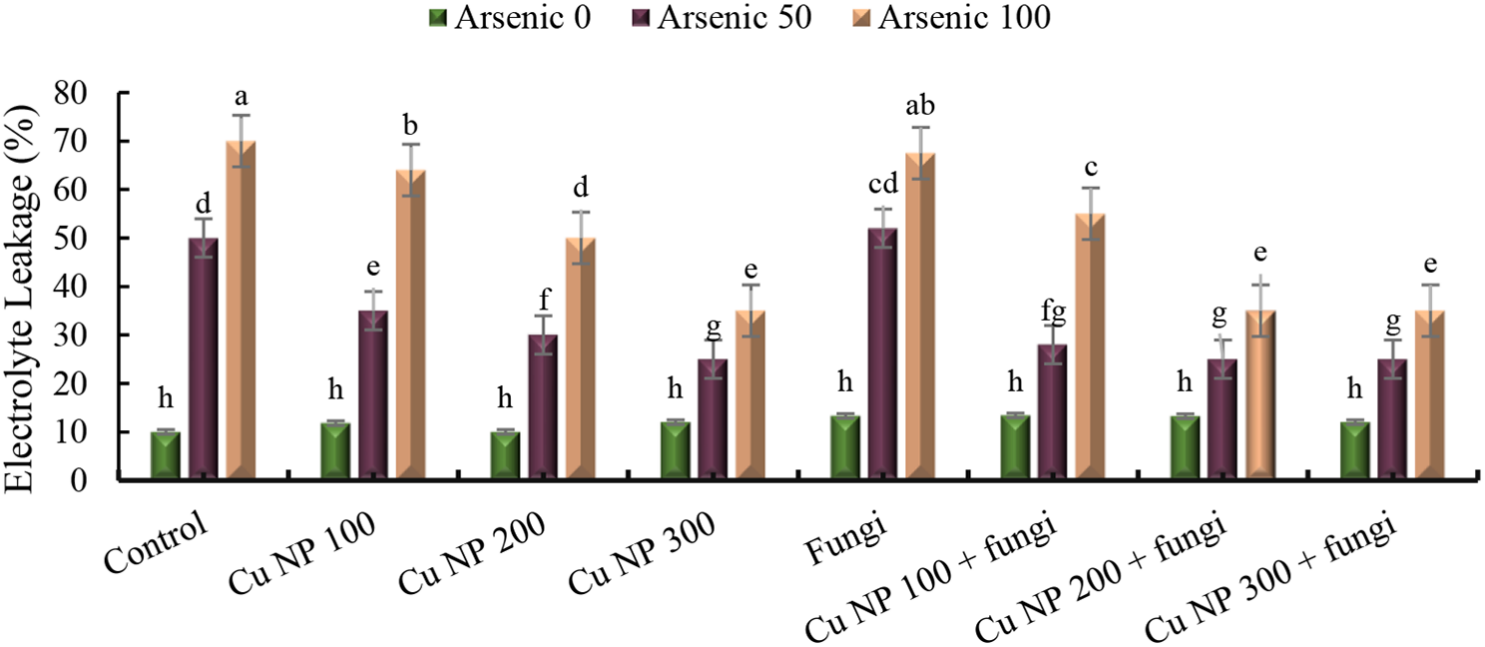
Effect of CuNPs and mycorrhiza association on electrolytic leakage of *Elymus sibiricus* under arsenic stress. Graph bars represents mean value of three replicates. Error bars shows standard error and different letters above bars obtained after Tukey’s HSD test, represents mean values are significantly different at *p* < 0.05

The table presents data on electrolyte leakage in different experimental groups subjected to various treatments. In the control group without stress, electrolyte leakage remains at 0%. However, when exposed to stressors like arsenic at 50 and 100 mg Kg^− 1^, the control group experiences significant increases in electrolyte leakage, reaching 400% and 600% respectively. Introducing copper nanoparticles at concentrations of 100 mM, 200 mM, and 300 mM shows varied effects. For instance, at 100 mM concentration, electrolyte leakage decreases to 18% when compared to the control, but in the presence of arsenic at 50 and 100 mg Kg^− 1^, it increases to 250% and 540% respectively. Similarly, at 200 mM concentration, the leakage remains at 0% in the control, but with arsenic, it increases to 200% and 400% respectively. The pattern continues with different concentrations of copper nanoparticles. Additionally, the inclusion of arbuscular mycorrhizal fungi demonstrates mixed outcomes. While the presence of these fungi alone or in combination with copper nanoparticles sometimes reduces electrolyte leakage, it significantly increases in the presence of arsenic, reaching up to >5 folds in some instances. These findings suggest complex interactions between stressors, nanoparticles, and fungi, influencing electrolyte leakage levels in the experimental groups (Fig. 6).

### Effect of CuNPs and mycorrhiza association on antioxidant enzymes activity in *E. sibiricus* under arsenic stress

#### Superoxide dismutase (SOD)

**Fig. 7:**
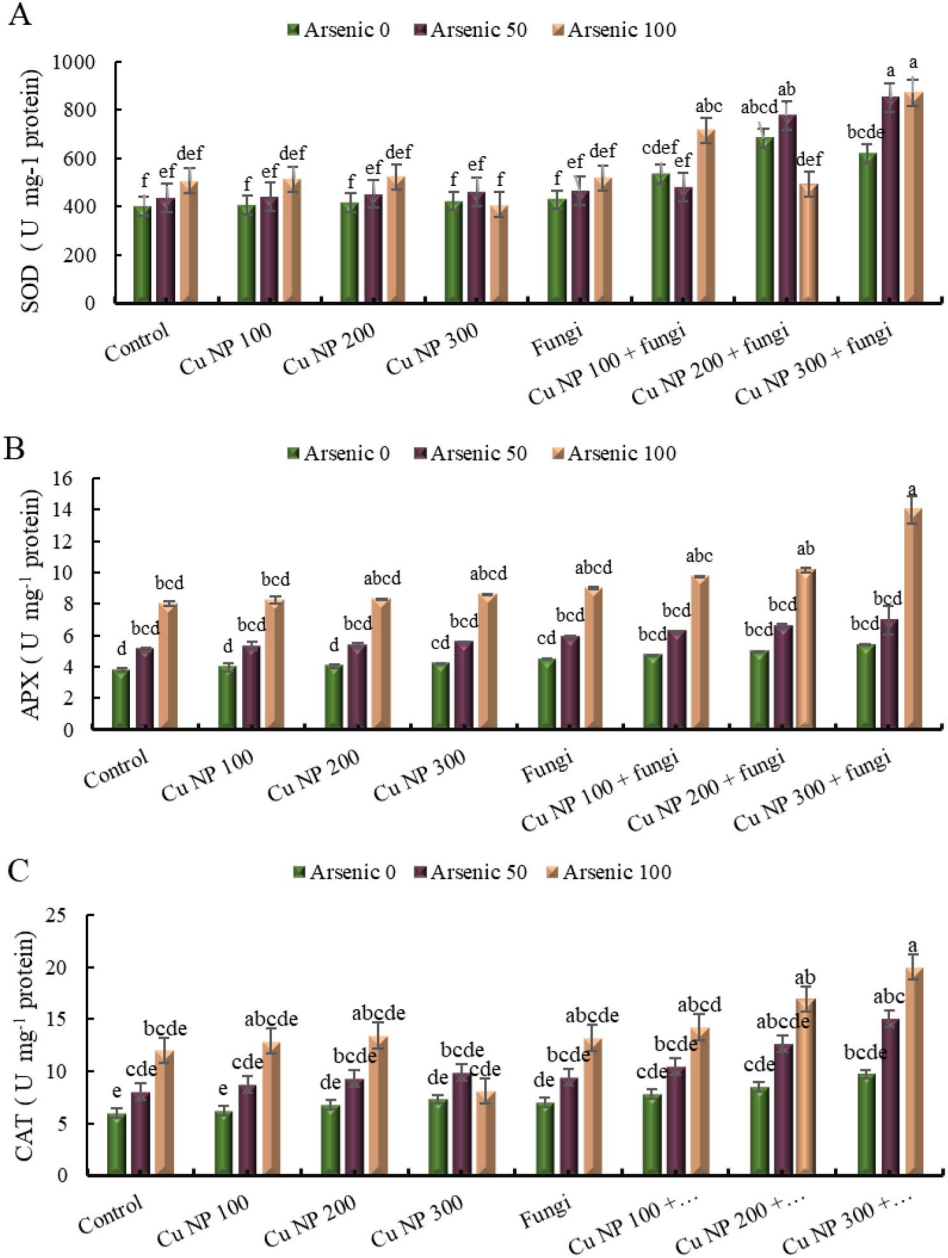
Effect of CuNPs and mycorrhiza association on activity of (**A**) superoxide dismutase, (**B**) ascorbate peroxidase and (**C**) catalase in *Elymus sibiricus* under arsenic stress. Graph bars represents mean value of three replicates. Error bars shows standard error and different letters above bars obtained after Tukey’s HSD test, represents mean values are significantly different at *p* < 0.05

The data provided presents the effects of different treatments on the stress levels (SOD activity) within a group. The control group without stress shows a baseline SOD activity of 0%. However, when subjected to stress alone (with 50 mg Kg^− 1^ or 100 mg Kg^− 1^ of arsenic), the SOD activity increases to 9% and 27% respectively. Introducing copper nanoparticles at 100 mM concentration in the control group results in a slight increase in SOD activity to 1%. When combined with arsenic stress (50 mg Kg^− 1^ or 100 mg Kg^− 1^), the SOD activity rises to 11% and 28% respectively. Similarly, copper nanoparticles at concentrations of 200 mM and 300 mM show similar trends when combined with arsenic stress. The introduction of arbuscular mycorrhizal fungi in the control group results in a moderate increase in SOD activity to 7%. When subjected to arsenic stress (50 mg Kg^− 1^ or 100 mg Kg^− 1^), the SOD activity further increases to 16% and 29% respectively. Combining arbuscular mycorrhizal fungi with copper nanoparticles at 100 mM concentration demonstrates a notable increase in SOD activity to 34%, while with arsenic stress (50 mg Kg^− 1^) the activity drops to 20%, but significantly rises to 79% with 100 mg Kg^− 1^ of arsenic stress. Similar trends are observed with arbuscular mycorrhizal fungi combined with copper nanoparticles at 200 mM and 300 mM concentrations. These findings suggest varying degrees of modulation in SOD activity based on the combinations of stressors and treatments applied (Fig. 7A).

#### Ascorbate peroxidase (APX)

The data presented shows the effects of different treatments on a group denoted as “APX.” The control group, without any additional stressors, exhibits a 0% change. However, when subjected to stressors such as arsenic at concentrations of 50 and 100 mg Kg^− 1^, the control groups show increases in activity by 34% and 110% respectively. Introducing copper nanoparticles at concentrations of 100 mM, 200 mM, and 300 mM in the control group yields changes of 5%, 6%, and 11% respectively. When arsenic was added alongside copper nanoparticles at these concentrations, the activity increases further, with the highest enhancement observed at 100 mM and 100 mg Kg^− 1^ of arsenic, showing a 127% increase. Additionally, the presence of arbuscular mycorrhizal fungi alone results in a 18% increase, which significantly amplifies with the addition of arsenic, especially at higher concentrations, reaching up to 137%. Interestingly, combining arbuscular mycorrhizal fungi with copper nanoparticles shows even greater effects, with the highest increase observed at 300 mM concentration, reaching a staggering 268% when exposed to 100 mg Kg^− 1^ of arsenic. These findings suggest complex interactions between different treatments and stressors, indicating potential avenues for further research into stress mitigation strategies (Fig. 7B).

#### Catalase (CAT)

The data presented illustrates the effects of different treatments on a group, denoted as “CAT.” The control group, which experiences no stress, shows a baseline of 0%. However, when subjected to stress, indicated by the addition of arsenic at 50 mg Kg^− 1^, there’s an increase in the response to 33%, and a further increase to 100% at 100 mg Kg^− 1^. Introducing copper nanoparticles at 100 mM in the control group shows a response of 3%, which rises to 45% with arsenic at 50 mg Kg^− 1^ and remarkably to 115% with arsenic at 100 mg Kg^− 1^. Similarly, the introduction of copper nanoparticles at concentrations of 200 mM and 300 mM also displays varying responses, with and without arsenic stress. Additionally, the presence of arbuscular mycorrhizal fungi alone in the control group exhibits a response of 17%, which significantly increases to 120% with arsenic at 100 mg Kg^− 1^. Furthermore, combinations of arbuscular mycorrhizal fungi with copper nanoparticles at different concentrations under various arsenic stress conditions depict diverse responses, with some exceeding 100%, indicating a significant enhancement compared to individual treatments (Fig. 7C).

#### Glutathione reductase (GR)

**Fig. 8:**
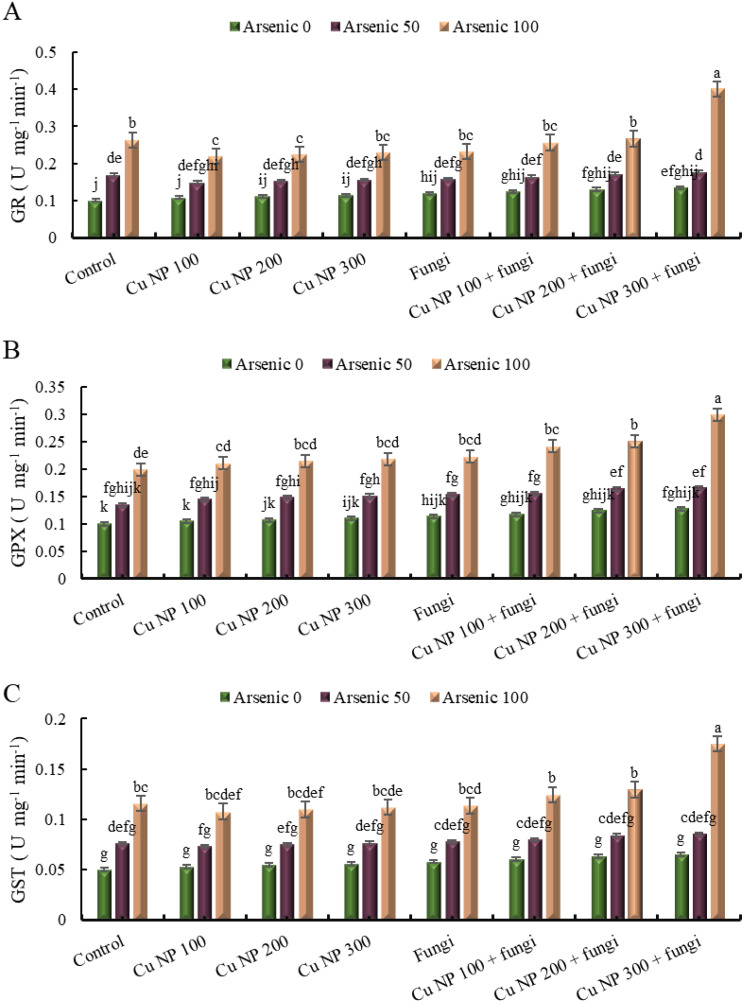
Effect of CuNPs and mycorrhiza association on activity of (**A**) glutathione reductase, (**B**) glutathione peroxidase and (**C**) glutathione transferase in *Elymus sibiricus* under arsenic stress. Graph bars represents mean value of three replicates. Error bars shows standard error and different letters above bars obtained after Tukey’s HSD test, represents mean values are significantly different at *p* < 0.05

The data provided outlines the activity levels of Glutathione Reductase under various experimental conditions. The control group, representing normal conditions without stress, demonstrates a baseline activity of 0%. When subjected to increasing doses of copper nanoparticles (100 mM, 200 mM, and 300 mM), there was a noticeable decrease in enzyme activity compared to the control, with activity levels ranging from 8 to 14%. However, when arsenic was introduced alongside copper nanoparticles, there’s a significant variation in enzyme activity depending on the dosage. For instance, at 100 mg Kg^− 1^ of arsenic alongside copper nanoparticles, the enzyme activity ranges from 49 to 129%. Moreover, the introduction of arbuscular mycorrhizal fungi also affects enzyme activity, with varying degrees of impact depending on the presence of arsenic and copper nanoparticles. In some instances, the combination of arbuscular mycorrhizal fungi with arsenic and copper nanoparticles leads to a notable increase in enzyme activity, exceeding even in the control levels, particularly evident at 100 mg Kg^− 1^ of arsenic with 300 mM of copper nanoparticles, where the enzyme activity reaches >3 folds. This data suggests complex interactions between these factors, highlighting potential avenues for further study into their combined effects on enzyme activity (Fig. 8A).

#### Glutathione peroxidase (GPX)

The data presented shows the activity of Glutathione peroxidase under various conditions. In a control setting without stress, the activity level was recorded as 0%. However, when subjected to increasing concentrations of copper nanoparticles, there’s a noticeable increase in activity, with 100 mg Kg^− 1^ concentration showing the highest activity at 100%. Interestingly, the addition of arsenic alongside copper nanoparticles further enhances the activity, surpassing even the maximum activity observed with copper nanoparticles alone. This trend was consistent across different concentrations of copper nanoparticles. Additionally, the involvement of arbuscular mycorrhizal fungi further amplifies Glutathione peroxidase activity, especially when combined with arsenic and copper nanoparticles, demonstrating synergistic effects. Overall, these findings highlight the complex interactions between nanoparticles, heavy metals, and biological agents, shedding light on potential strategies for mitigating oxidative stress (Fig. 8B).

#### Glutathione transferases (GST)

The data provided pertains to the activity levels of Glutathione transferases under various experimental conditions. When operating without stress (control without stress), the activity level was recorded as 0%. However, when subjected to different doses of stress inducers such as Arsenic and copper nanoparticles, the activity levels vary. For instance, in the control group treated with 50 mg Kg^− 1^ of Arsenic, there was a 52% increase in activity compared to the unstressed control. This increment rises to 131% with a higher dose of 100 mg Kg^− 1^ Arsenic. Similarly, the introduction of copper nanoparticles at different concentrations also affects the activity of Glutathione transferases. For example, with 100 mM copper nanoparticles, the activity level in the control group was 6%, which increases to 47% and 115% respectively when exposed to Arsenic at 50 and 100 mg Kg^− 1^. The pattern continues with varying concentrations of copper nanoparticles, indicating a complex interplay between stressors and the activity of Glutathione transferases. Moreover, the inclusion of arbuscular mycorrhizal fungi in the experimental setup also influences the enzyme activity, with different combinations of stressors showing varied effects. Overall, the data illustrates the modulation of Glutathione transferase activity in response to different stressors and their combinations, highlighting the intricate nature of stress response mechanisms in biological systems (Fig. 8C).

### Effect of CuNPs and mycorrhiza association on non-enzymatic antioxidants in *E. sibiricus* under arsenic stress

#### Ascorbic acid (AsA)

**Fig. 9:**
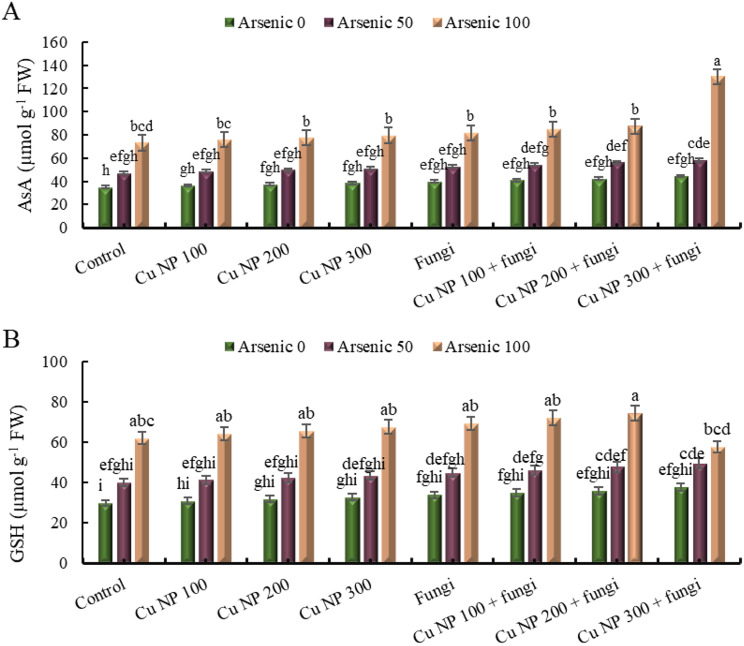
Effect of CuNPs and mycorrhiza association on non-enzymatic antioxidants (**A**) ascorbic acid and (**B**) glutathione content in *Elymus sibiricus* under arsenic stress. Graph bars represents mean value of three replicates. Error bars shows standard error and different letters above bars obtained after Tukey’s HSD test, represents mean values are significantly different at *p* < 0.05

The data provided represents the levels of ascorbic acid under various experimental conditions. The control group, without any stressors, shows a baseline of 0%. When subjected to increasing doses of ascorbic acid (50 and 100 mg Kg^− 1^), the levels rise to 35% and 109%, respectively. Introducing copper nanoparticles at different concentrations (100 mM, 200 mM, and 300 mM) in the control group results in varying levels of ascorbic acid, ranging from 4 to 10%. When arsenic was added along with copper nanoparticles, the levels of ascorbic acid increase further, with the highest level recorded at 127%. Additionally, the presence of arbuscular mycorrhizal fungi alone increases the ascorbic acid level to 14%, and in combination with arsenic and/or copper nanoparticles, the levels show an incremental rise, reaching up to 271%. These findings suggest complex interactions between different factors, including metals, nanoparticles, and fungi, in influencing the levels of ascorbic acid (Fig. 9A).

#### Glutathione (GSH)

Glutathione contents were elevated under 50 (35%) and 100 mg Kg^− 1^ (109%) arsenic contaminated soil (Fig. 9B). Further, application of CuNPs and mycorrhizal soil amendments boosted the glutathione level in control as well as As-stress plants. The maximum glutathione increment was found in plants grown in arsenic contaminated soil (100 mg Kg^− 1^) treated with CuNPs foliar spray (200 mM) along with mycorrhizal soil amendments.

### Effect of CuNPs and mycorrhiza association on arsenic (As) uptake in roots and translocation to shoot in *E. sibiricus*

**Fig. 10:**
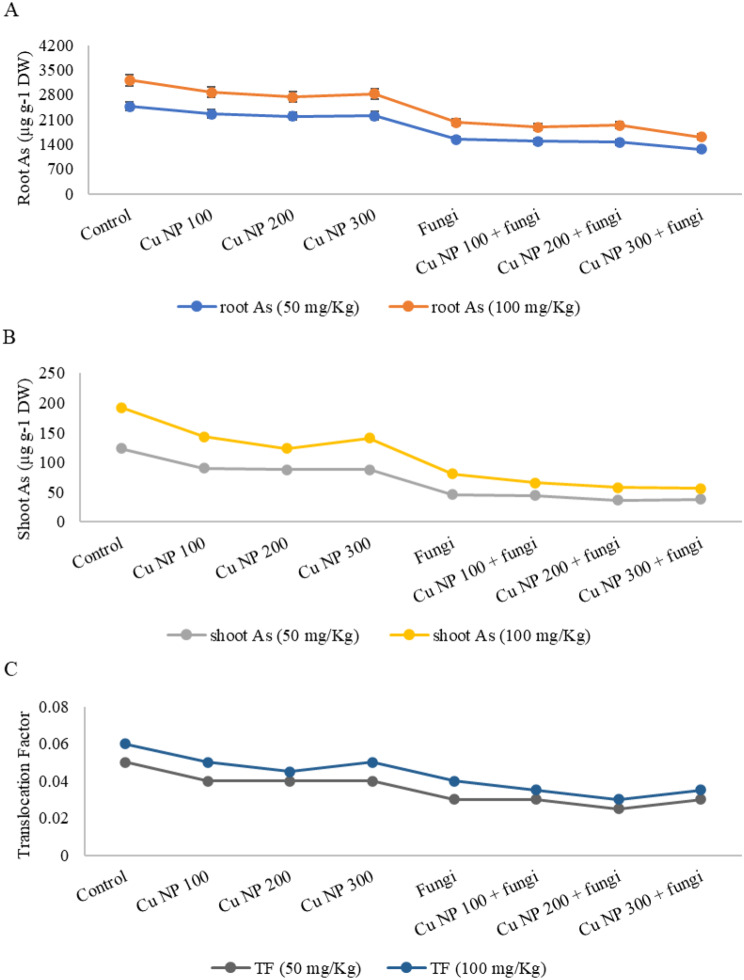
Effect of CuNPs and mycorrhiza association on (**A**) As uptake in root (**B**) As uptake in shoot and (**C**) translocation factor in *Elymus sibiricus* under arsenic stress. Values presented in the graphs are average of three replicates

Elevated level of As was detected in roots and shoot tissues of *E. sibiricus* plants subjected to 50 and 100 mg Kg^− 1^ arsenic polluted soil. As depicted in Fig. 10A-C, As uptake in roots and its subsequent transfer to shoot was significantly limited with foliar spray of CuNPs (100, 200 and 300 mM) alone or in synergistic application with mycorrhizal inoculation. The combined action of CuNPs and fungal inoculation was more effective in limiting As uptake in roots and its translocation towards shoot. This was also evident from translocation factor in Fig. 10C, that foliar treatment of CuNPs and mycorrhizal amendments limited As heavy metals in the soil or in the roots of *E. sibiricus* plants.

### Pearson’s correlation and Principle component analysis

**Fig. 11:**
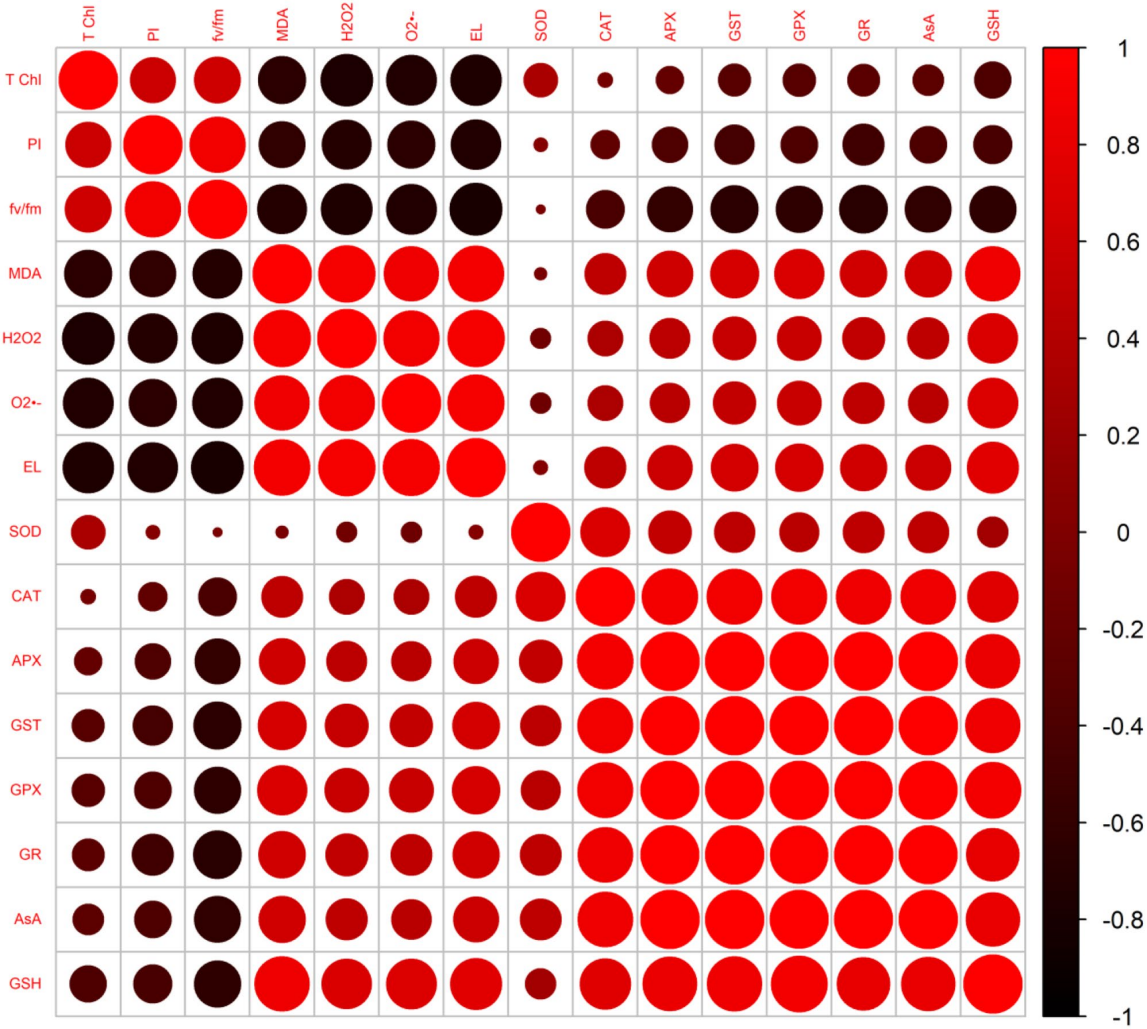
Pearson’s correlation to assess the effect of CuNPs and mycorrhiza association on various attributes of *Elymus sibiricus* under arsenic stress. Various abbreviations used are; T chl = total chlorophyll, PI = performance index, fv/fm = quantum efficiency of PSII, MDA = malondialdehyde, H_2_O_2_ = hydrogen peroxide, O_2_^•‾^ = superoxide anion, EL = electrolytic leakage, SOD = superoxide dismutase, CAT = catalase, APX = ascorbate peroxidase, GST = glutathione transferase, GPX = glutathione peroxidase, GR = glutathione reductase, AsA = ascorbic acid and GSH = glutathione

Figure 11 showed correlation among various attributes of *E. sibiricus* studied in this research. Photosynthesis related parameters (T chl, f_v_/f_m_ and PI_ABS_) had significant negative correlation with stress markers and enzymatic and non-enzymatic antioxidants. Level of stress markers and antioxidants was directly influenced by oxidative stress due to arsenic heavy metal. This depicted that arsenic toxicity resulted in oxidative stress in *E. sibiricus* plants which ultimately reduced photosynthesis and related parameters.

**Fig. 12:**
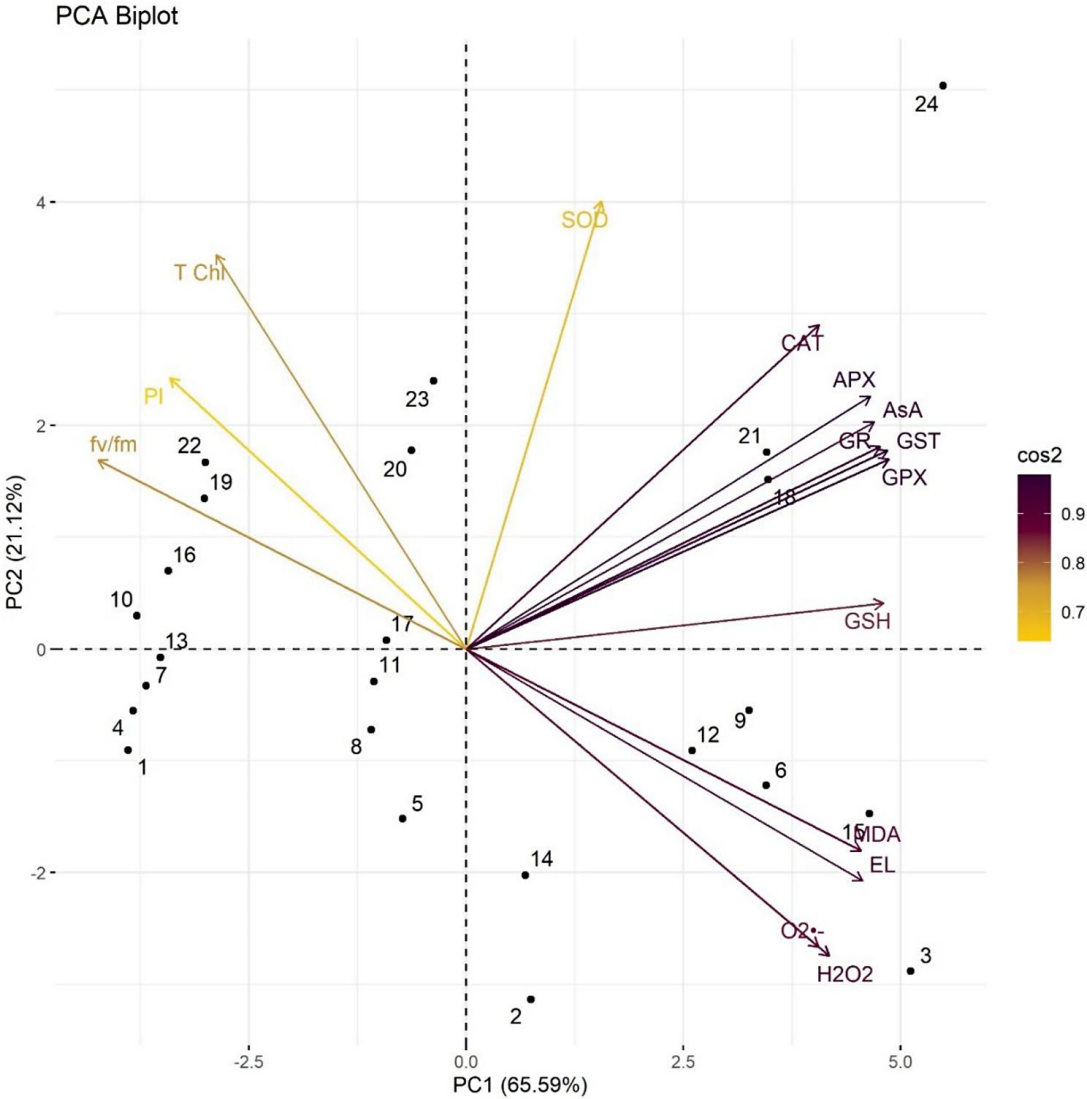
Principle component analysis (PCA biplot; dots represents PCA of individual treatment while arrows represents PCA of all parameters) to assess the effect of CuNPs and mycorrhiza association on various attributes of *Elymus sibiricus* under arsenic stress. (Various abbreviations used are same as in Fig. 11

PCA biplot in Fig. 12, showed that all the individual treatments and studied parameters of *E. sibiricus* were significantly distributed in first two principle components i.e. PC1 contributing 65.59% and PC2 contributing 21.12%. All the parameters studied in this research can be separated inn two groups, first group aligned with PC1 and the second grouped aligned with PC2. The parameters aligned with PC2 had significant negative correlation with those aligned to PC1. Arrows at same distance from the origin points reflected that these parameters are highly interconnected.

## Discussion

The increased level of heavy metal contaminants in arable areas exert injurious effects on physiochemical activities, growth, yield and biomass production of crop plants [56]. Heavy metal stress decreases root formation and growth which decline uptake and translocation of water and nutrients leading to stunted plant growth [57]. During the current research, significant reduction in shoot and root growth of *E. sibiricus* plants was observed in case of As spiked soils. Arsenic induced phytotoxicity elevated ROS synthesis, extended cellular cycles, injured root and shoot tissues, and abridged plant efficiency to uptake minerals and water [58, 59]. Besides, As stress declined establishment of AMF with applied *E. sibiricus* plants especially in case of treatments devoid of CuNPs. Yet, slight development of metal resistant hyphae in *E. sibiricus* in plant roots exert positive influence on growth of the plants. Hence, the separate and combined application of CuNPs and AMF mitigated As toxicity verified by improved plant growth. Reduction in the synthesis of ROS, regulation of carbohydrates production and nutrition improvement due to CuNPs and/ or AMF supplementation enhanced plant growth. Nevertheless, plants treated with CuNPs in presence of AMF exhibited more pronounced effects as compared to separate applications of CuNPs or AMF. The increased uptake and translocation of Cu besides growth promoting attributes of AMF enable plants to tolerate As stress [60, 61].

Metal stress declines chlorophyll synthesis, disturbs electron transport chain, impedes photosystem II (PSII) and interrupts Calvin cycle [62]. Our findings are in agreement with [63, 64], who reported reduction in the biosynthesis of metal stressed *Oryza sativa*; *Lolium perenne* plants respectively. The decreased biosynthesis of photosynthetic contents in stressed plants results in diminution of photosynthesis rate [65]. Similarly, As stress diminished the activity of chlorophyll synthesizing enzymes [66]. The increased synthesis of ROS perhaps damaged chloroplast structure, chlorophyll protein complex and enhanced the activity of chlorophyllase in As stressed plants [67]. [68] also documented negative effect As-induced stress on electron transport chain, photosynthetic content, photosystem II (PSII), gas exchange parameters and Calvin cycle [69] Hence, As stress reduces the efficiency of carbon transport and assimilation [70, 71]. [72] also confirmed that As presence in guard cells affects stomatal conductivity and reduces carbon dioxide transportation towards chloroplasts [71]. In the same way, the replacement of As with essential mineral nutrients related to biosynthesis of photosynthetic pigments reduced photosynthesis in *E. sibiricus* plants growing in As contaminated media. Though, improvement in the antioxidant system of plants receiving CuNPs and/ or AMF treatment/s significantly mitigated As toxicity and increased biosynthesis of photosynthetic pigments. By the way, the combination of CuNPs and AMF was more effective to enhance mineral nutrition and promote chlorophyll synthesis as compared to their individual application. Higher uptake and translocation of essential mineral cations elevates synthesis of photosynthetic pigments [73]. Alternatively, As deteriorates the ultrastructure of chloroplast, reduces transpiration rate, and declines stomatal conductivity. The improvement in carbon assimilation, stomatal movement, photosynthetic activity, and chlorophyll content in As stressed plants may be credited to CuNPs and AMF [72, 74].

Plants alleviate metal toxicity by enhancing the biosynthesis of phenolics and flavonoids having aptitude to act as ROS scavengers, phytochelatins and make complex with heavy metals [75, 76]. Our data exhibited higher flavonoids and phenolic content in *E. sibiricus* plants subjected to As stress. Possibly, As toxicity increased the bioactivity of phenylalanine ammonialyase, a crucial enzyme of phenylalanine pathway involved in the synthesis of flavonoids and phenolic compounds [77]. Moreover, thioredoxin activation in stressed plants synthesized higher levels of anthocyanins to mitigate As toxicity [78]. [79] also reported that the increased synthesis of flavonoids and phenolic compounds in metal stressed *Oryza sativa* plants alleviate stress.

The overproduction of ROS causes oxidative injuries in metal stressed plants [80]. Correspondingly, the higher activity of LOX besides elevated level of ROS enhances lipid peroxidation in metal stressed plants [81]. During this study, *E. sibiricus* plants having higher LOX and increased biosynthesis of ROS exhibited intensification of MDA content under As stress. Contrary to this, CuNPs and/ or AMF treated plants growing in As polluted soils, showed reduction in MDA which may be attributed to the diminished synthesis of ROS and reduced activity of LOX. It was also noted that superior carboxylation efficacy and maximum reduction in MDA level was demonstrated by As stressed plants receiving simultaneous application of CuNPs and AMF. The reduction in MDA level and higher photosynthetic activity was also a result of improved antioxidant system, modulated synthesis of GSH and phytohormones in CuNPs and AMF treated plants subjected to As regimes [82]. [83] also observed that in CuNPs and/ or AMF maintained membranous integrity through reducing MDA synthesis and enhancing antioxidative activity.

Enhancement of antioxidant activities enable plants to mitigate stress [84]. Antioxidant enzymes including CAT and SOD enhance plant stress tolerance by detoxifying ROS. Though, metal toxicity decreases the antioxidative potential of CAT and SOD [85]. Our data displayed that initially CAT activity increased following decrease inclination, demonstrating that As negatively affected the antioxidative machinery. Yet, during the current study, the upregulated activity of SOD in As stressed *E. sibiricus* presented a cumulative tendency [86]. While reduction in lipid peroxidation along with increased H_2_O_2_ scavenging due to higher antioxidative activity of CAT and SOD confirm stress amelioration in *E. sibiricus* plants receiving joint application of CuNPs and AMF. Additionally, detoxification of ROS through GSH-related GST and GPX enzymes defend cell components from oxidative injuries [87]. The increased level of H_2_O_2_ and inadequate ROS scavenging caused by higher metal concentration may lower GPX and GST activity. Particularly, the combined treatment of CuNPs and AMF improved the activities of GST and GPX in As stressed plants was associated with the modulations of GSH level [88, 89]. Therefore, it was assumed that CuNPs and AMF augmented the activity of GST and GPX and induced stress tolerance against As stress through enhancement of ROS scavenging by GSH [90].

The antioxidant enzymes DHAR and MDHAR intercede in the regeneration of AsA [91]. While, antioxidant GSH was connected to the renaissance of ascorbic acid (AsA) in the AsA − GSH cycle and assuages oxidative injuries in plants. While, GR and GST assist in the retrieval of GSH from GSSG. The decrease of AsA content and amplification in the biosynthesis of GSH, GSSG and DHA in As- stressed *E. sibiricus* plants revealed the conciliation of the redox system [92, 93]. Higher GSH content in As-stressed plants designate that GSH may not be sufficient to scavenge ROS level with inadequate amount of AsA [94]. For this reason, it was perceived that the higher level of ROS besides reduced activity of APX possibly decreased AsA level in plants subjected to As stress [95]. The increased activity of DHAR, MDHAR and GR recycled GSH and AsA to regulate redox balance in plants receiving co-application of CuNPs and AMF As contaminated [96]. The reduced level of oxidative stress in CuNPs and AMF treated plants was arbitrated to the regulation of appropriate GSH and AsA pools [97].

Methylglyoxal (MG) was a mutagen causing cellular injuries [98]. The increased biosynthesis of MG in metal stress conditions decreases physiological and metabolic activities of plants leading to decreased growth and development [99, 100]. In the presence of reduced GSH, *lactoylglutathione* lyase (Gly I), the first enzyme of the glyoxalase system converts MG to *S-*d-lactoylglutathione (SLG) [101]. While, the second enzyme of glyoxalase system, glyoxalase II also known as Gly II, catalyzes the hydrolysis of *S*-D-lactoylglutathione to form glutathione and lactic acid [102, 103]. The combined activity of glyoxalase enzymes assists in the detoxification of MG through transforming it into a non-injurious form while employing GSH as a cofactor [104]. So, the glyoxalase defense system plays an important role to alleviate metal phytotoxicity through detoxification of MG content [105]. However, the higher level of MG in As-stressed plants during the current study showed the inability of the glyoxalase defense system to alleviate metal stress. Probably, the activity of Gly I and Gly II was not sufficient to detoxify the level of MG synthesized by the stressed plants [7]. However, AMF inoculation in combination with CuNPs significantly decreased MG level through enhancing the catalytic activity of Gly I and Gly II in As-stressed plants [106, 107].

The study of gene expression level correlated the respective enzymatic activity. Additionally, higher gene expression detoxified more amount of ROS to alleviate As stress [78]. Very few studies have evaluated the effect of metal toxicity on gene expression [108, 109]. According to our information, no study has documented the expression level of antioxidant enzymes related genes CuNPs and AMF treated *E. sibiricus* plants under As stress. The current research evidently advocates that supplementation of CuNPs or AMF mitigated As induced phytotoxicity in *E. sibiricus* by decreasing ROS synthesis and increasing the antioxidative activity of defense related enzymes.

Higher As concentration enhances As content in plants growing in contaminated soil [110, 111]. [112] observed lower metal content in above ground parts of plants owing to the immobilization of metal in root tissues by well-organized mycorrhizal symbiotic fixation. Our results exhibited that AMF assisted *E. sibiricus* abridged As uptake and translocation in the shoots, nonetheless, amplified As concentrations in the roots. [113] found that AMF restricted the *Triticum aestivum* in the mycorrhizosphere zone and hindered the translocation of metal ions to plant shoots. In another study, AMF diminished As content in shoot and root of upland rice [114]. The decreased As content in the shoot of AMF applied plants may be credited to the hyphae of AMF which act as an As-pool to avert its translocation towards shoots through binding, chelating, and adsorbing. Additionally, the dilution effects connected to an augmented biomass and a declined As distribution to shoot of the plants decreased As content in AMF inoculated plants [115, 116].

## Conclusion

In summary, the combined application of CuNPs or AMF increased As stress tolerance in *E. sibiricus* through detoxification of ROS and MG besides improvement of antioxidative system. Supplementation of CuNPs or AMF improved antioxidant defense machinery through improving the antioxidative activity as well as transcription level of genes involved in plant stress tolerance. Furthermore, reduction in As uptake and translocation, diminution in oxidative injuries and improvement in photosynthetic activity were credited to the stress ameliorative potential of CuNPs or AMF in *E. sibiricus* under As contaminated environments. Correspondingly, the extent of metal stress tolerance exerted in *E. sibiricus* was associated with the variations in the modulations triggered in As tolerance pathways through CuNPs and AMF application. Our results advocate that CuNPs and AMF combination improve plant growth and nutrition, leading to the formation of novel fungicide/ biofertilizer. Still, further field research seems mandatory to elucidate the advantageous effects of CuNPs and AMF treatments on other members of Poaceae family subjected to heavy metal stress.

**Table 1:** List of chemicals and reagents used for the synthesis of copper nanoparticles

Sr. no.	Chemical composition	Molar mass (g/mol)	% Purity	Distributor
1.	CuSO_4_.5H_2_O	250	99%	DAEJUNG
2.	NaOH	40	99%	DAEJUNG

**Table 2:** Energy-dispersive X-ray (EDX) spectroscopy of Cu and CuO nanoparticles

Elemental analysis weight by weight%	
Compounds	Percentage
Copper	53.12%
Oxygen	34.81%
Sulphur	6.31%
Carbon	2.21%
Zinc	3.55%

**Table 3:** Effect of CuNPs and Mycorrhiza association on shoot length of *Elymus sibiricus* under arsenic stress

Shoot length			
Treatments	As 0 mg/Kg soil	As 50 mg/Kg soil	As 100 mg/Kg soil
Control	22.95±1.1475	16.06±0.803	14.34±0.717
CuNPs 100	28.11±1.4055	21.21±1.4055	17.34±1.0605
CuNPs 200	32.13±0.867	24.32±1.6065	22.26±1.216
CuNPs 300	35.32±1.113	31.55±1.766	22.95±1.5775
AMF	32.7±1.1475	20.65±1.635	17.78±1.0325
CuNPs 100 + AMF	34.44±0.889	29.83±1.722	26.96±1.4915
CuNPs 200 + AMF	36.03±1.348	34.29±1.8015	32.86±1.7145
CuNPs 300 + AMF	38.76±1.643	34.42±1.938	30.41±1.721

Values presented in the table are mean of three replicates ± standard errors

**Table 4:** Effect of CuNPs and Mycorrhiza association on root length of *Elymus sibiricus* under arsenic stress

Root length			
Treatments	As 0 mg/Kg soil	As 50 mg/Kg soil	As 100 mg/Kg soil
Control	7.80±0.390	5.46±0.273	4.87±0.243
CuNPs 100	9.55±0.477	7.21±0.477	5.89±0.360
CuNPs 200	10.92±0.294	8.26±0.546	7.56±0.413
CuNPs 300	12.00±0.378	10.72±0.600	7.80±0.536
AMF	11.11±0.390	7.02±0.555	6.04±0.351
CuNPs 100 + AMF	11.70±0.302	10.14±0.585	9.16±0.507
CuNPs 200 + AMF	12.25±0.458	11.65±0.612	11.17±0.582
CuNPs 300 + AMF	13.17±0.558	11.70±0.658	10.33±0.585

Values presented in the table are mean of three replicates ± standard errors

## Data Availability

The data sets associated with present work are available from the corresponding authors on reasonable request.
